# Exercise Training Combined with *Bifidobacterium Longum* OLP-01 Supplementation Improves Exercise Physiological Adaption and Performance

**DOI:** 10.3390/nu12041145

**Published:** 2020-04-19

**Authors:** Wen-Ching Huang, Yi-Ju Hsu, Chi-Chang Huang, Hsuan-Chen Liu, Mon-Chien Lee

**Affiliations:** 1 Department of Exercise and Health Science, National Taipei University of Nursing and Health Sciences, Taipei 11219, Taiwan; 2 Graduate Institute of Sports Science, National Taiwan Sport University, Taoyuan 33301, Taiwan

**Keywords:** probiotic, training, energy balance, OLP-01, fatigue, SCFAs

## Abstract

Probiotics exert multiple health benefits, including gastrointestinal health, immunoregulation, and metabolic disease improvement, by modulating microbiota to maintain eubiosis via the hypothalamic–pituitary–adrenal (HPA) and brain–gut–microbiome axes. Physiological fatigue, mental stress, and gastrointestinal discomfort under the demands of athletic performance as well as immunosuppression are common during endurance training and competition. Limited studies investigated the functional effects of probiotic supplementation on endurance training. *Bifidobacterium longum* subsp. *Longum* OLP-01 (OLP-01), isolated from an elite Olympic athlete, was combined with a six-week exercise training program with gradually increasing intensity. In this study, Institute of Cancer Research (ICR) mice were assigned to sedentary, exercise, OLP-01, or exercise + OLP-01 groups and administered probiotic and/or treadmill exercise training for six weeks to assess exercise performance, physiological adaption, and related metabolites. The exercise + OLP-01 group demonstrated higher performance in terms of endurance and grip strength, as well as improved fatigue-associated indexes (lactate, ammonia, creatine kinase (CK), lactate dehydrogenase (LDH), and glycogen content), compared with the other groups. OLP-01 supplementation significantly ameliorated inflammation and injury indexes (platelet/lymphocyte ratio (PLR), aminotransferase (AST), and CK) caused by prolonged endurance exercise test. Moreover, acetate, propionate, and butyrate levels were significantly higher in the exercise + OLP-01 group than in the sedentary and OLP-01 groups. Athletes often experience psychological and physiological stress caused by programed intensive exercise, competition, and off-site training, often leading to poor exercise performance and gastrointestinal issues. Functional OLP-01 probiotics are considered to be a nutritional strategy for improving physiological adaption, oxidative stress, inflammation, and energy balance to ensure high physical performance. Based on these results, probiotics combined with exercise training is a potential strategy for ensuring high physical performance of athletes, which should be further investigated through microbiota validation.

## 1. Introduction

Regular and optimized physical activities can be considered not only prevention but also therapy for different chronic diseases and related complications [1]. Exercise, particularly aerobics, also significantly improves physiological adaption by enhancing cardiovascular capacity and energy metabolites [2]. The balance between intensive exercise-induced oxidative stress and the antioxidant system play an important role in athlete health and in the prevention of molecular and cellular damage [3]. Studies elucidated that functional ingredients, including vitamin C, vitamin E, green tea extract, and quercetin, regulate oxidative stress and tissue damage in athletes [4,5]. Excessive exogenous antioxidants may have detrimental effects on physiological adaption, and antioxidants from a varied and balanced diet may be the best approach for the maintenance of optimal antioxidant status [6]. In addition, a study reported that exercise increases microflora diversity and the number of beneficial microbial species and commensal bacteria with health benefits [7], and microbiota improve exercise performance through antioxidative enzymes, as revealed through gnotobiotic animal model validation [8]. Microbiota also regulate oxidative stress and inflammatory responses, as well as modulate metabolism and energy expenditure during intense exercise [9].

Probiotics were demonstrated to have functional effects on gastrointestinal (GI) conditions, including diarrhea, inflammatory bowel disease (IBD), and liver disease, through microbiota composition modulation [10]. Metabolic conditions (obesity and diabetes) can also be managed by probiotic and synbiotic supplementation from fermented food sources [11]. Fermented milk, containing *Lactobacillus delbrueckii* subsp. bulgaricus and *Streptococcus thermophilus*, improved the behavior of aged mice as well as their redox state and immune cell function [12]. A mixture of probiotics also improved the recovery of intestinal multibarriers in DSS-induced colitis by rebuilding the structure and diversity of gut microbiota [13]. Our previous clinical and animal studies reported that *L. plantarum* TWK10, isolated from Taiwanese pickled cabbage, exerted beneficial effects on body composition, energy production, and physiological adaption [14,15]. In triathletes, *L. plantarum* PS128 ameliorated inflammation, oxidative stress, fatigue, and injury-related biochemical indexes induced by intensive training [16]. Thus, probiotics exert various functional effects, leading to health benefits and health promotion.

*Bifidobacterium longum* OLP-01 isolated from the gut microbiota of a weightlifting gold medalist showed the beneficial effects of fatigue mitigation and increased energy production [17]. The exercise training was well-known for its benefits toward physiological adaption, and the probiotics were also able to exert multiple bioactivities. In this study, we hypothesized that OLP-01 combined with exercise intervention would facilitate exercise physiological adaption and performance. The aim was to reveal the different effects of probiotics on exercise training and, in particular, inform athletes regarding nutritional strategies.

## 2. Materials and Methods

### 2.1. Probiotics

*B. longum* subsp. *Longum* OLP-01 (OLP-01) was selected and isolated from an elite weightlifting Olympic gold medalist. The strain was also further identified by an independent third party, the Food Industry Research and Development Institute (Hsinchu, Taiwan). OLP-01 was cultivated and maintained by Glac Biotech Co., Ltd. (Tainan, Taiwan), and lyophilized powder was applied at a concentration of 1.07 × 10^11^ CFU/g, equivalent to about 1 × 10^9^ CFU/day for humans. Before supplementation, all aliquots of lyophilized powder were stored in a −20 ℃ refrigerator and prepared in sterilized saline buffer for daily use. The dose of OLP-01 used in current animal study (i.e., 1.03 × 10^10^ CFU/kg) was based on that used in a previous study [17]; OLP-01 was administered for 6 weeks via oral gavage. The sedentary and exercise treatment groups received the same volume of saline according to individual body weight.

### 2.2. Experimental Design

**Figure 1:**
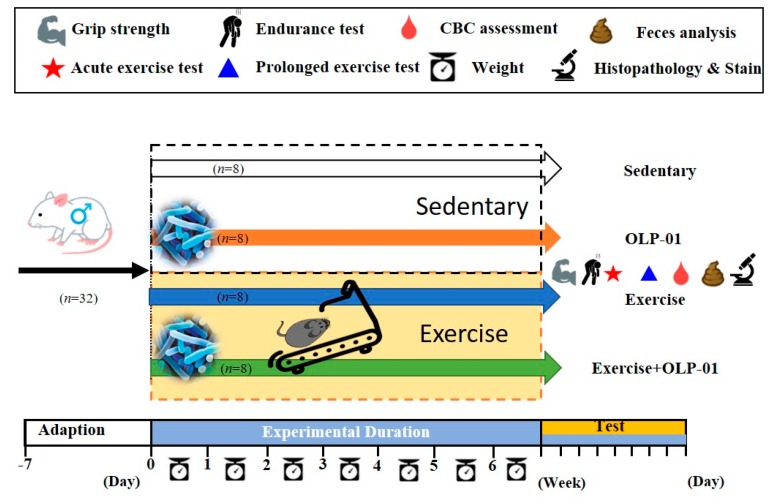
Experimental design to examine the effects of exercise and probiotics on exercise adaption. The animals were randomly assigned to the indicated four groups (sedentary, OLP-01, exercise, and exercise + OLP-01). Physical fitness and related assessments were conducted during the test.

Institute of Cancer Research (ICR) strain mice (5 weeks old), purchased from BioLASCO (Yi-Lan, Taiwan) and reared under specific pathogen-free (SPF) conditions, were used in this study. During the experimental duration, all animals were provided sufficient chow diet (No. 5001; PMI Nutrition International, Brentwood, MO, USA) and sterilized water *ad libitum* and maintained on a 12 h light/dark cycle at 23 ℃ ± 2 ℃ and 50%–60% humidity. A veterinarian monitored the disease status and behavior of the animals. After 1 week of acclimation, the animals were randomly allocated into four groups (sedentary, exercise, OLP-01, and exercise + OLP-01). The animals had similar body weights at the beginning of the experiment and the indicated groups received the treadmill exercise protocol or/and probiotic supplementation for 6 weeks (Figure 1). After 6 weeks of intervention, physical fitness was evaluated by measuring forelimb grip strength and by conducting exhaustive swimming, and biochemical indexes were also determined in mice undergoing acute and prolonged exercise challenges. Blood, relevant tissues, and feces were also sampled to determine the complete blood count (CBC), body composition, glycogen, and metabolites, as well as for histology analysis. The Institutional Animal Care and Use Committee (IACUC) of National Taiwan Sport University approved all animal experiments in this study, and the study conformed to the guidelines of protocol IACUC-10801 approved by the IACUC ethics committee.

### 2.3. Aerobic Exercise Training

Mice underwent aerobic exercise training on a motor-driven treadmill (model MK-680; Muromachi Kikai, Tokyo, Japan) for 6 weeks, with motivation maintained through an electric shock grid under veterinarian surveillance. Mice in the exercise training groups (exercise and exercise + OLP-01) were initially acclimated to running at a speed of 10 m/min for 2 days prior to the training protocol. The training protocol began at a speed of 12 m/min and 35 min/day in the first week, and the speed was increased by 2 m/min every week until the sixth week (22 m/min). The slope was also elevated from 0% to 5% in the third week and maintained at 10% from the fourth to sixth weeks. The training protocol was slightly modified from a previous study protocol, and moderate exercise intensity corresponded to 65%–70% of maximal oxygen uptake [18].

### 2.4. Physical Activities

Physical activities included forelimb grip strength activities and exhaustive swimming, which were used to test anaerobic and aerobic capacities. Grip strength was assessed using a low-force testing system (Model-RX-5; Aikoh Engineering, Nagoya, Japan), with detailed procedures described previously [19]. To determine exercise performance, swimming to exhaustion was conducted to assess endurance capacity based on survival instinct. The animals were loaded with a weight equivalent to 5% of individual body weight and forced to swim in a tank until exhaustion. The time from the start of the experiment to exhaustion was recorded as the endurance index.

### 2.5. Peripheral Fatigue-Associated Biochemical Variables

Exercise-induced peripheral fatigue can be reflected by related biochemical indexes when evaluating physiological adaption. The acute exercise protocol included 15 min of swimming without any weight load; blood was sampled through submandibular collection at the beginning, immediately after 15 min of swimming, and during the subsequent 20 min rest interval for analysis of the indexes, such as lactate and ammonia. The prolonged exercise protocol included swimming for 90 min; blood was collected immediately after 60 min of rest and analyzed for blood urea nitrogen (BUN), creatine kinase (CK), and lactate dehydrogenase (LDH) levels. The blood samples were assessed using an autoanalyzer (Hitachi 7060; Hitachi, Tokyo, Japan) and a CBC analyzer.

### 2.6. Short Chain Fatty Acid Analysis

Feces samples were obtained 1 day before euthanization. They were weighed, suspended in 1 mL of water with 0.5% phosphoric acid per 0.1 g of sample, and stored in −30 °C immediately after collection until homogenization. Feces were homogenized for 2 min and centrifuged for 10 min at 14.8 RPM. The supernatant was isolated for ethyl acetate (300 μL) extraction, and the organic phase was collected for Agilent 5977B GC-MS (Agilent Technologies; Palo Alto, CA, USA) analysis. The GC instrument was fitted with a Nukol™ Capillary GC Column (30 m × 0.25 mm id, 0.25 μm df) and helium was used as the gas carrier, injected at 1 mL/min. The column temperature was 90 °C initially and then increased to 150 °C at 15 °C/min, 170 °C at 5 °C/min, and finally to 250 °C at 20 °C/min; this temperature was maintained for 2 min (total time of 14 min). Solvent delay was 3.5 min. The detector was operated in the electron impact ionization mode (electron energy of 70 eV), with scanning conducted in the 30–250 m/z range. The temperatures of the ion source, quadrupole, and interface were 230 °C, 150 °C, and 280 °C, respectively. Short-chain fatty acids (SCFAs) were identified based on the retention time of standard compounds and with the assistance of the NIST 08 and Wiley7N libraries.

### 2.7. Clinical Biochemical Profiles

After 95% CO_2_ asphyxiation euthanization of mice, blood samples were immediately collected through cardiac puncture and the sera were separated through centrifugation at 1000× *g* for 15 min at 4 °C after complete clotting for analysis of clinical biochemical variables, including aspartate aminotransferase (AST), alanine transaminase (ALT), CK, glucose (GLU), blood urea nitrogen (BUN), creatinine (CREA), uric acid (UA), albumin (ALB), albumin (ALB), total cholesterol (TC), and total protein (TP), using an autoanalyzer (Hitachi 7060, Hitachi, Tokyo, Japan).

### 2.8. Body Composition, Histology, and Glycogen Analysis

The important visceral organs, including the heart, liver, kidney, spleen, muscle (gastrocnemius and soleus), and cecum, alongside perirenal fat (white adipocyte tissue), were accurately excised and weighed after sacrifice and dissection for determining body composition. Then, the organs were preserved in 10% formalin for further paraffin-embedded procedures. Indicated tissue sections (4 μm) were collected from paraffin blocks and immersed in xylene and alcohol, followed by hematoxylin for 3 min and counterstaining with eosin for 1 min. Parts of the muscle and liver samples were kept in liquid nitrogen for glycogen content analysis. The analysis protocol was modified from a previous study; detailed procedures were as described previously [20].

### 2.9. Statistical Analysis

Data were represented as the mean ± SD. Statistically significant differences in physical activity, biochemistry, lactate, body weight, body composition, growth curve, and glycogen content between the groups were analyzed using one-way and two-way analysis of variance (ANOVA), followed by multiple comparisons with post-hoc Tukey’s test. Data were considered statistically significant when the probability of a type I error was less than 0.05.

## 3. Results

### 3.1. Effects of Exercise and Probiotic Intervention on Growth Curve and Body Composition

**Figure 2:**
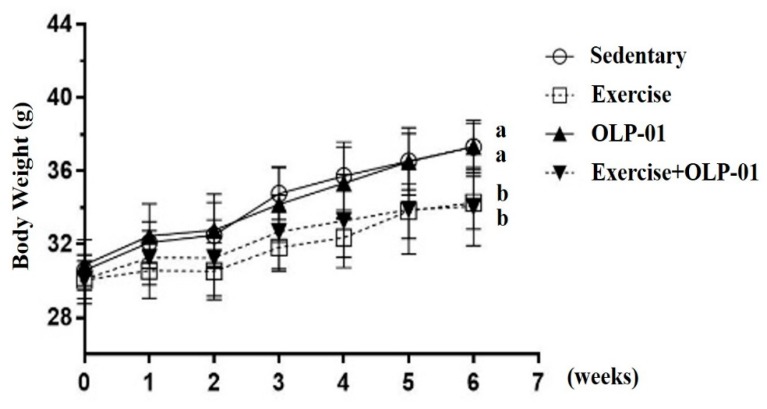
Effects of six weeks of exercise and probiotic interventions on the growth curve. Data are expressed as the mean ± SD per group. Bars with different letters (a, b) were significantly different at *p* < 0.05.

At the beginning of the experiment, the indicated four groups had a weight of approximately 31 g, with no significant differences found among the groups (F(3,28) = 0.433, *p* = 0.731). The weights of the indicated groups were continuously monitored during the six weeks of exercise and/or probiotic supplementation. A significant main effect was observed for exercise (F(1,28) = 32.6, *p* < 0.0001), but no probiotic or interaction main effects were observed (F(1,28) = 0.55, *p* = 0.816; F(1,28) = 0.78, *p* = 0.782, respectively). A significant difference in body weight was observed among the groups (F(3,28) = 10.9, *p* < 0.0001) in the sixth week. The body weights of the exercise and exercise + OLP-01 groups were significantly lower than those of the sedentary and OLP-01 groups by approximately 8.5% (*p* < 0.05) (Figure 2). Therefore, the exercise training effects could be directly reflected in growth curves, and the possible physiological effects were further validated by the following experimental designs.

Body composition was measured by assessing the weights of individual representative tissues or organs, including the heart, liver, spleen, muscle (gastrocnemius and soleus), kidney, and cecum, alongside perirenal fat. A significant difference was found in perirenal fat (F(3,28) = 4.54, *p* = 0.01) among the groups, which was significantly lower in the exercise and exercise + OLP-01 groups than in the sedentary and OLP-01 groups (*p* < 0.05) (Table 1). Moreover, no significant differences were found in the weights of the other tissues or dietary intake among the groups (*p* > 0.05).

### 3.2. Effects of Exercise and Probiotics on the Performance of Exhaustive Exercise

**Figure 3:**
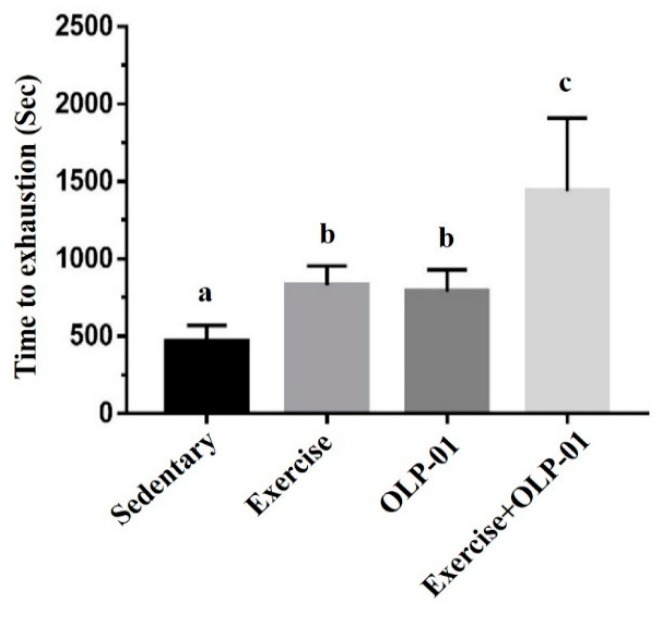
Effects of six weeks of exercise and probiotic interventions on exhaustive swimming time. Columns with different letters (a, b, c) were significantly different at *p* < 0.05.

The performance of exhaustive exercise demonstrated the endurance or aerobic capacity of mice subjected to the intervention. Endurance capacities were not significantly different between the groups before the experiment (data not shown). After six weeks of administration, the results showed a significant difference among the groups (F(3,28) = 13.1, *p* < 0.0001). The exhaustive swimming time was significantly higher in the exercise, OLP-01, and exercise + OLP-01 groups than in the sedentary group (*p* = 0.03, *p* = 0.038, and *p* < 0.0001, respectively), and exhaustive swimming time was also significantly elevated in the exercise + OLP-01 group than in the exercise and OLP-01 groups (*p* = 0.001 and *p* < 0.0001, respectively) (Figure 3). The results demonstrated a significant difference in the exercise and OLP-01 main effects (F(1,28) = 30.1, *p* < 0.001; F(1,28) = 28.3, *p* < 0.001, respectively) and the interaction effects (F(1,28) = 4.32, *p* = 0.047).

### 3.3. Effects of Exercise and Probiotic Intervention on Forelimb Grip Strength

**Figure 4:**
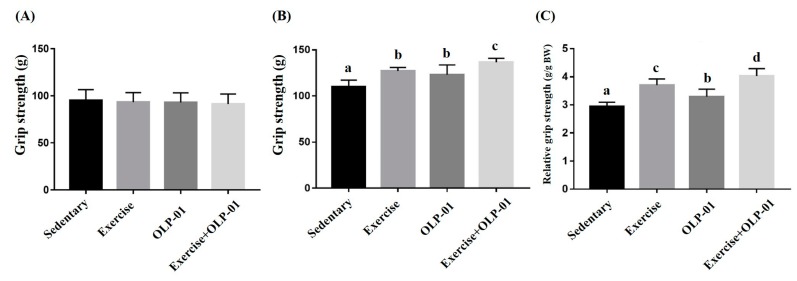
Effects of six weeks of exercise and probiotic interventions on baseline grip strength (**A**), after administration (**B**), and relative strength (**C**). Data are represented as mean ± SD and the columns with different letters (a, b, c, d) were significantly different at *p* < 0.05.

A previous study showed that grip strength was positively and significantly correlated with anthropometric factors, including age, weight, and body mass index [21]. Grip strength and relative grip strength calibrated by individual body weight is demonstrated in Figure 4. In this study, the baseline of grip strength was not significantly different between the groups before administration (F(3, 28) =0.732, *p* = 0.547) (Figure 4A). After six weeks of intervention, significant differences were observed in absolute strength (F(3, 28) = 19.42, *p* < 0.0001) and relative strength (F(3, 28) = 34.3, *p* < 0.0001) among the groups. The exercise, OLP-01, and exercise + OLP-01 groups showed significantly higher grip strength than the sedentary group (*p* < 0.001), and the exercise + OLP-01 group also showed significantly elevated grip strength compared with the exercise and OLP-01 groups (*p* = 0.013 and *p* < 0.001, respectively) (Figure 4B). Similar trends were found for relative grip strength (Figure 4C), but the exercise group showed significantly higher relative grip strength than the OLP-01 group. A significant difference was observed in the exercise and OLP-01 main effects (F(1, 28) = 19.6, *p* < 0.0001 and F(1, 28) = 18.3, *p* < 0.0001, respectively), but no interaction effect was observed (F(1, 28) = 1.41, *p* = 0.254).

### 3.4. Effects of Exercise and Probiotic Intervention on Fatigue-Associated Biochemistry

Several biochemical indexes, such as lactate, ammonia, CK, and LDH, are highly associated with exercise duration and intensity because of energy metabolites and oxidative stress. As shown in Table 2, the acute exercise challenge rapidly reflected energy demands and energy metabolites, such as lactate. Significant differences were observed in lactate concentration (F(3, 28) = 13.0, *p* < 0.0001) and lactate production rate (F(3, 28) = 22.9, *p* < 0.0001) immediately after exercise. The exercise, OLP-01, and exercise + OLP-01 groups showed significantly lower lactate production than the sedentary group, and the exercise + OLP-01 group also showed significantly lower lactate production than the exercise and OLP-01 groups post-exercise. Regarding self-comparison indexes, the lactate production rate demonstrated more significant benefits in the exercise + OLP-01 group than the other three groups (F(3, 28) = 22.9, *p* < 0.0001), but no significant difference was observed in the clearance rate among the groups (F(3, 28) = 0.174, *p* = 0.913).

**Figure 5:**
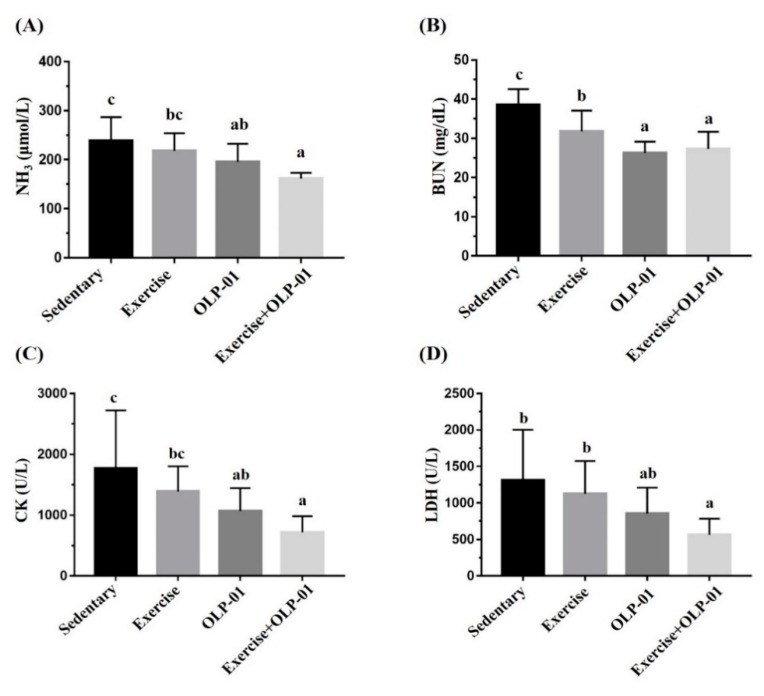
Effects of six weeks of exercise and probiotic interventions on ammonia (**A**), blood urea nitrogen (BUN) (**B**), CK (**C**), and LDH (**D**) levels after acute and prolonged exercise challenges. Data are represented as the mean ± SD, and columns with different letters (a, b, c) were significantly different at *p* < 0.05.

Ammonia produced by exercise was also evaluated using direct NH_3_ measurement and related metabolic BUN indexes. As shown in Figure 5A, the ammonia concentrations of the OLP-01 and exercise + OLP-01 groups were significantly lower than that of the sedentary group (*p* = 0.025 and *p* < 0.0001, respectively), and the exercise + OLP-01 group also showed a significantly lower ammonia concentration than the exercise group (*p* = 0.004). Ammonia can be further metabolized as BUN through the urea cycle and removed by the kidneys as urine. Significant differences were also found in the BUN index among the groups (F(3, 28) = 13.9, *p* < 0.0001), with BUN significantly decreasing in the OLP-01 and exercise + OLP-01 groups compared to the sedentary and exercise groups (Figure 5B). Intensive exercise induced significant oxidative stress and increased CK and LDH markers, which are indices of tissue damage. As shown in Figure 5C, the CK index demonstrated significant differences among the groups (F(3, 28) = 4.9, *p* = 0.007), and the OLP-01 and exercise + OLP-01 groups showed significantly decreased prolonged exercise-induced CK levels compared with the sedentary group. CK levels in the exercise + OLP-01 group were also significantly lower than those in the exercise group (*p* = 0.026). The LDH index (Figure 5D) also demonstrated a significant difference among the groups (F(3, 28) = 3.98, *p* = 0.017), with the LDH level was significantly ameliorated in the exercise + OLP-01 group compared to the sedentary and exercise groups (*p* = 0.003 and *p* = 0.021, respectively).

### 3.5. Effects of Exercise and Probiotic Intervention on Clinical Biochemistry and CBC

At the end of experiment, mice in the indicated groups were sacrificed to evaluate their clinical biochemistry. As depicted in Table 3, the AST and CK indexes exhibited significant differences among the groups. Both the AST and CK indexes in the exercise group were significantly higher than those in the sedentary, OLP-01, and exercise + OLP-01 groups (*p* < 0.05). No significant differences were found between the groups in the other indexes, namely, ALT, CLU, CREA, BUN, UA, TC, TG, ALB, and TP. No significant difference was observed in CBC in terms of white blood cells (WBCs) and related types of WBCs (neutrophil, lymphocyte, monocytes, and basophils), but the platelet and platelet/lymphocyte ratio (PLR) showed significant differences among the groups (F(3, 28) = 14.1, *p* < 0.0001; F(3, 28) = 17.0, *p* < 0.0001, respectively). Platelet and PLR indexes were significantly higher in the exercise group than in the sedentary, OLP-01, and exercise + OLP-01 groups (*p* < 0.05), and exercise training-caused platelet and PLR increases were significantly ameliorated in the exercise + OLP-01 group (*p* < 0.0001) (Table 4).

### 3.6. Effects of Exercise and Probiotic Intervention on Tissue Glycogen Content

**Figure 6:**
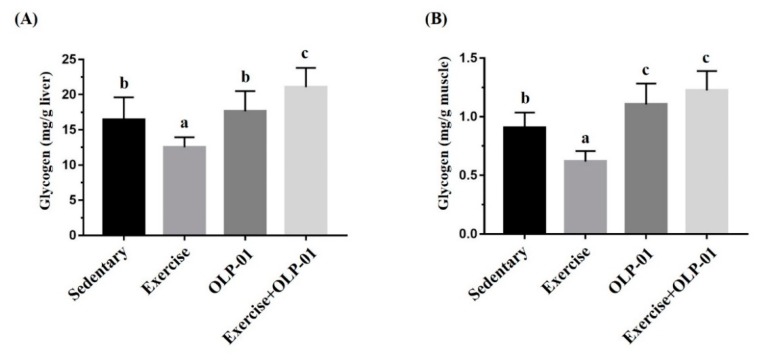
Effects of six weeks of exercise and probiotic interventions on liver (**A**) and muscle (**B**) glycogen contents. The four groups underwent 90 min of swimming exercise, and blood was sampled after 60 min of rest. Data are represented as the mean ± SD, and columns with different letters (a, b, c) were significantly different at *p* < 0.05.

Glycogen contents in the liver and muscle (gastrocnemius and soleus tissues) were measured using the chemical reaction method, i.e., by the colorimetric formation of glycogen–iodine complexes. Significant differences were observed in the liver and muscular tissues (F(3, 28) = 15.63, *p* < 0.0001; F(3, 28) = 26.67, *p* < 0.0001, respectively) among the groups (Figure 6). The glycogen content in liver tissue significantly decreased in the exercise group compared to the sedentary control group, and the exercise + OLP-01 group also demonstrated a significantly higher glycogen content than the sedentary, exercise, and OLP-01 groups (Figure 6A). The glycogen content decrease caused by exercise was also observed in the muscle tissue, and the OLP-01 and exercise + OLP-01 groups showed significantly higher glycogen content than the sedentary and exercise groups (Figure 6B).

### 3.7. Effects of Exercise and Probiotics on Histology

**Figure 7:**
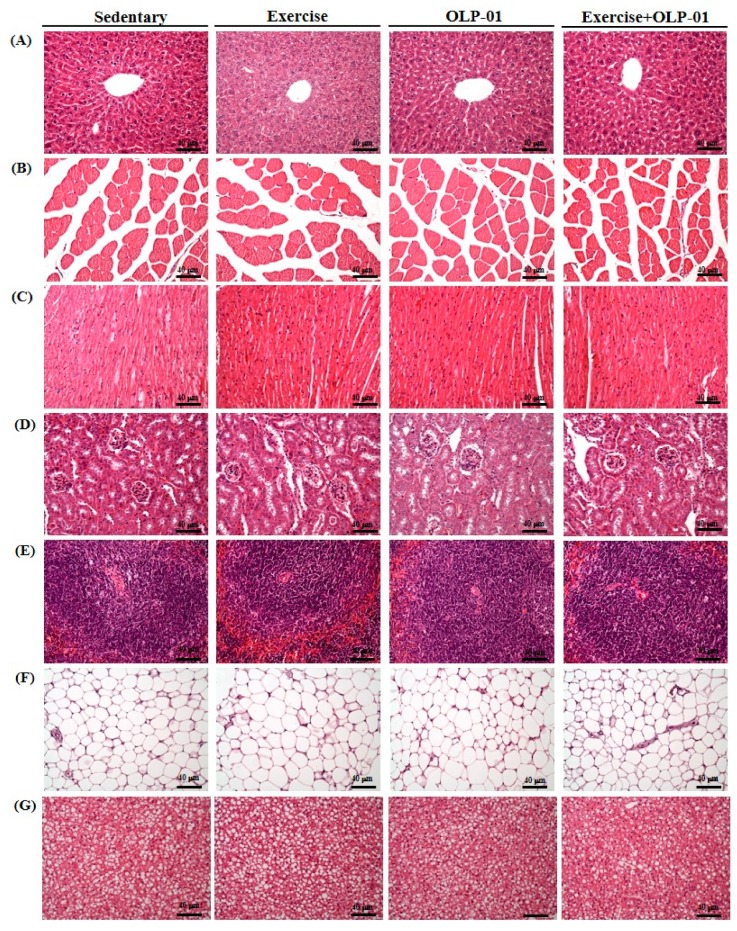
Effects of six weeks of exercise and probiotic interventions on histopathology. (**A**) Liver, (**B**) muscle, (**C**) heart, (**D**) kidney, (**E**) spleen, (**F**) WAT, and (**G**) BAT tissue in mice. Specimens were photographed using a light microscope (H & E staining, magnification: 200×; bar, 20 μm). WAT, white adipocyte tissue; BAT, brown adipocyte tissue; muscle: gastrocnemius and soleus tissues.

Figure 7 shows the results of the evaluation of potential pathological changes in different tissues (liver, muscle, heart, kidney, spleen, WAT, and BAT) after programed exercise training and probiotic supplementation. In the liver, the arrangement of sinusoid and hepatic cords did not significantly change among the groups after the indicated treatments. Moreover, Zenker’s degeneration, hyperplasia, and inflammatory cell infiltration were not observed in muscular tissues (cardiomyocytes and skeletal tissues). In the kidney, the structure of the renal tubules and glomeruli did not differ among the groups. Focal apoptosis in white pulp and extramedullary hematopoiesis in the spleen were not observed in the groups. The perirenal fat pad (white adipose tissue) was composed of adipocytes, which are very large cells with small, uniform nuclei usually located near the plasma membrane. BAT adipocytes were histologically observed to be polygonal with acidophilic multivacuolated and granular cytoplasm. The nuclei of BAT adipocytes remained in the center and multiple droplets exhibited an appearance of tiny soap bubbles within the cell.

### 3.8. Effects of Exercise and Probiotic Intervention on SCFAs

Feces were used to analyze SCFAs, including acetate, propionate, isobutyrate, butyrate, and valeric acid (Table 5). The exercise + OLP-01 group showed significantly higher acetate, propionate, and butyrate levels than the sedentary and OLP-01 groups. SCFAs in the OLP-01 group did not significantly differ from those in the sedentary and exercise groups, but the exercise-only group also demonstrated a significant increase in butyrate concentration compared with the sedentary group.

## 4. Discussion

In the current study, *B. longum* OLP-01 isolated from an elite weightlifting athlete was combined with regular aerobic treadmill exercise training for six weeks to evaluate physiological effects and performance. Results demonstrated that *B. longum* OLP-01 combined with exercise training (the exercise + OLP-01 group) synergistically increased endurance capacity compared with training and probiotic-alone treatments (exercise and OLP-01 groups, respectively). Moreover, *B. longum* OLP-01 also significantly improved exercise-associated peripheral fatigue indexes and ameliorated oxidative stress-related injury indexes, possibly through inflammation regulation. From the perspective of ergogenic aid and health promotion, these probiotics could be further investigated in regard to functional activities in sport science. 

The physiological effects of exercise are contingent on exercise intensity, type, and duration as well as energy demands, oxidative stress, and cardiovascular and metabolic adaptations [2]. During exercise, central and peripheral fatigue can occur, alongside alterations in endocrine function, immune function, systemic inflammation, and oxidative stress [22,23]. Peripheral fatigue can be evaluated using biomarkers related to energy metabolites, such as lactate, ammonia, and BUN, with these indexes reflecting ATP-generation efficiency and the associated metabolism during exercise. Oxidative stress caused by exercise results in reactive oxygen species (ROS) production with inadequate electron transfer through the mitochondrial respiratory chain, which is related to increased oxygen consumption for the energy demands of muscular contraction [24]. ROS production is caused by the oxidation of proteins and lipids and is accompanied by a marked decrease in antioxidant capacity and an increased risk of tissue injury [25]. Therefore, antioxidant and injury indexes, including glutathione, glutathione peroxidase, catalase, total antioxidant capacity, LDH, CK, AST, and ALT, were applied in previous studies on ergogenic acids [26,27]. In a previous study [17], OLP-01 was found to improve peripheral fatigue-associated indexes (NH_3_, BUN, and CK) after acute exercise challenges without training intervention; these indexes were also improved by OLP-01-only treatment in the present study, with OLP-01 exerting similar physiological bioactivities after at least five weeks of supplementation. This study demonstrated that OLP-01 combined with training (exercise + OLP-01 group) significantly improved these indexes induced by exercise challenge and training.

Exercise training modulated physiological adaption and improved exercise performance. In a previous study, four weeks of aerobic swimming training significantly increased endurance capacity but not the lactate and BUN indexes [28], and training intensity (six weeks of training with load that was 2% of body weight) was a critical factor for lactate and ammonia metabolism adaption in another study [29]. In this study, exercise training (exercise group) with the treadmill protocol with gradually increasing intensity not only elevated endurance performance but also increased the lactate and BUN metabolic indexes, thereby providing physiological benefits (Table 2 and Figure 5B). Pyruvate oxidation was enhanced by the exercise training-induced increase in pyruvate dehydrogenase activity, thereby reducing lactate production and accumulation during exercise [30]. In addition, exercise-induced ammonia elevation was further metabolized as BUN through the urea cycle and removed by the kidneys as urine, which are considered to be fatigue biomarkers of the energy and metabolism balance. Exercise training significantly reduced creatinine and BUN levels, providing benefits regarding renal activities [31]; citrulline is also considered an ergogenic aid from the perspective of ammonia metabolism [32]. In this study, exercise training improved the modulation of BUN and ammonia metabolism, and the exercise + OLP-01 group showed significantly higher physiological adaption than the exercise group (Figure 5).

Oxidative stress generated from excessive free radical production leads to inflammation and tissue injury during intensive exercise. Exercise training elevates antioxidant capacity and ameliorates oxidative stress to maintain a redox balance [33]. The optimized exercise training intensity induces the production of reactive oxygen and nitrogen species, thereby improving physiological adaption and exerting nonlinear/hormetic effects. Excessive exogenous antioxidant supplementation interferes with the ROS/RNS signaling pathway for favorable muscle adaptation [34,35]. Exercise training, regardless of the intensity, volume, and type of exercise and the target population, was reported to maintain the redox state balance and to exert health-related benefits [36]. Regarding the antioxidant supplementation strategy, natural antioxidant nutrients/ingredients from a balanced and diverse diet may synergistically optimize antioxidant effects in conditions with high oxidative stress, such as intensive training [37]. Moreover, probiotics exhibit antioxidant capacity through multiple possible mechanisms, including chelation, the production of antioxidant metabolites, the regulation of host antioxidant signaling pathways, and the modulation of host microbiota [38]. Therefore, OLP-01 probiotics, in combination with exercise, could be considered as a nutritional supplementation strategy to improve physiological adaption-associated oxidative stress and inflammation.

Athletes typically participating in highly intensive training and competition, such as those participating in marathons and triathlons, may exhibit immunosuppression and inflammation, eventually leading to illness, including upper respiration infection and GI problems [39]. Moreover, strenuous and prolonged exercise may cause splanchnic ischemia and dysregulation of the intestinal tight junction barrier, thereby increasing permeability, which may lead to local and systemic inflammation [40]. In human and animal studies, PLR and NLR were elucidated as appropriate and potential indexes for systemic inflammation [41,42]. In this study, exercise training significantly elevated PLR as a systemic inflammation index, and OLP-01 supplementation ameliorated the effects of exercise training to maintain homeostasis (Table 4). Supplementation with multiple probiotic strains could therefore provide benefits on intestinal permeability, the immune system, intestinal microbiota, and inflammation and could also mitigate respiratory tract infections and GI symptoms for ensuring the overall health of athletes [43].

Glycogen is an important energy source for meeting the energy demands and stability of physical activities. The intensity and duration of exercise training significantly affects the glycogen content, and carbohydrate availability may be a critical strategy for glycogenesis, with effects on higher performance and adaption [44]. Studies also showed that exercise training upregulated metabolic enzymes and transport for higher glycogen bioavailability and replenishment rate [45]. Butyrate, an SCFA, maintains blood glucose homeostasis and promotes glycogen metabolism through the GPR43-AKT-GSK3 signaling pathway [46]. In this study, the exercise + OLP-01 group exhibited significantly elevated SCFAs, particularly acetate, propionate, and butyrate, compared with the sedentary and OLP-01 groups (Table 5). A related study also demonstrated that probiotics (*L. acidophilus*) regulated glycogen synthesis-related genes (GSK-3β and Akt) and glycogen content in tissues [47]. In addition, the effects of bacteria-derived molecules and metabolites on the host immune system were shown to include regulation of gene expression for indicated functional activities [48]. Therefore, OLP-01 supplementation also possibly regulated glucose metabolism, oxidative stress, and inflammation through other mechanisms, an area which needs further validation. Therefore, SCFAs may not be the only regulators that modulate glycogen synthesis in regard to OLP-01 supplementation.

A previous study elucidated that feces acetate content levels were associated with plasma acetate levels and that acetate restored the endurance capacity of antibiotic-treated animals, possibly by serving as an important energy substrate during exercise [49,50]. In elite athletes, the indicated microbiota upregulated genes in a major pathway metabolizing lactate to propionate, which had a higher relative abundance post-exercise, as revealed by shotgun metagenomic analysis [51]. The microbiome elucidates important roles in regard to endurance exercise by producing SCFAs [50]. In addition, SCFAs also exert protective effects on the intestinal barrier function by inhibiting NLRP3 inflammasomes and autophagy to ensure intestinal permeability and health [52]. Therefore, OLP-01 supplementation improved lactate metabolism after acute exercise without increasing SCFAs production. These data regarding the OLP-01 group indicated that there were other mechanisms at play from the exercise and OLP-01 group.

Therefore, OLP-01 supplementation possibly exerts functional effects through the modulation of beneficial physiological adaptions and the production of related metabolites, particularly when combined with exercise training. Exercise combined with OLP-01 probiotics demonstrated functional benefits on physical activities through modulation of inflammation and physiological adaption. The microbiota represented possible regulators of functional activities, but this area should be further investigated. Athletes often experience intestinal discomfort because of high-intensity exercise, psychological stress, and off-site training. Thus, appropriate probiotics could be considered to be an alternative nutritional strategy for athletes to improve both their physiological adaption and their exercise performance.

## 5. Conclusions

In this study, OLP-01 probiotic supplementation combined with exercise training synergistically increased exercise performance and improved physiological adaption. OLP-01 and/or exercise training also beneficially modulated physiological adaption via different mechanisms, which should be validated further in future work. The microbiota composition changes caused by exercise and probiotics should be also further studied and analyzed in regard to appropriate modulation of physiological adaptions. In current study, probiotics demonstrated multiple functional effects on health and exercise performance promotion. OLP-01 could be considered to be an ergogenic aid, particularly in athletes undergoing regular intensive training.

## Figures and Tables

**Table 1:** Effects of exercise and probiotics on body composition and diet intake.

Characteristic	Sedentary	Exercise	OLP-01	Exercise + OLP-01
Liver (g)	2.13 ± 0.29	2.08 ± 0.20	2.12 ± 0.28	2.13 ± 0.25
Muscle (g)	0.36 ± 0.04	0.34 ± 0.03	0.36 ± 0.04	0.35 ± 0.03
Kidney (g)	0.65 ± 0.08	0.66 ± 0.07	0.66 ± 0.09	0.65 ± 0.04
Heart (g)	0.20 ± 0.03	0.21 ± 0.02	0.21 ± 0.03	0.20 ± 0.03
Spleen (g)	0.26 ± 0.03	0.25 ± 0.11	0.26 ± 0.06	0.25 ± 0.06
Cecum (g)	0.72± 0.15	0.73± 0.11	0.69± 0.08	0.75± 0.18
Perirenal Fat (g)	0.124 ± 0.02 ^b^	0.094 ± 0.03 ^a^	0.123 ± 0.03 ^b^	0.086 ± 0.02 ^a^
Water intake (mL/mouse/day)	8.1 ± 0.9	8.2 ± 1.1	8.4 ± 1.2	8.3 ± 0.9
Diet intake (g/mouse/day)	7.5 ± 1.2	7.6 ± 1.5	7.7 ± 1.1	7.5± 1.3

Data are expressed as the mean ± SD in each group. Values in the same row with different superscript letters (^a^, ^b^) differed significantly at *p* < 0.05 by one-way analysis of variance (ANOVA). Muscle: gastrocnemius and soleus.

**Table 2:** The effects of exercise and probiotics on lactate metabolite profiles during acute exercise challenge.

Time Point	Sedentary	Exercise	OLP-01	Exercise + OLP-01
	Lactate (mmol/L)			
Before swimming (A)	2.3 ± 0.3 ^a^	2.3 ± 0.3 ^a^	2.4 ± 0.2 ^a^	2.4 ± 0.3 ^a^
After swimming (B)	6.8 ± 0.8 ^c^	6.2 ± 0.3 ^b^	6.1 ± 0.2 ^b^	5.2 ± 0.5 ^a^
After a 20 min rest(C)	3.9 ± 0.5 ^b^	3.7 ± 0.6 ^a,b^	3.3± 0.8 ^a,b^	3.1 ± 0.5 ^a^
**Rate of lactate production and clearance**				
Production rate = B/A	2.96 ± 0.2 ^c^	2.72 ± 0.3 ^b, c^	2.51 ± 0.2 ^b^	2.14 ± 0.1 ^a^
Clearance rate = (B − C)/B	0.43 ± 0.06	0.41 ± 0.09	0.45 ± 0.15	0.40 ± 0.06

Lactate metabolites were measured in the sedentary, exercise, OLP-01, and exercise + OLP-01 groups at three repeated time points. The lactate production rate was calculated as lactate production after exercise divided by lactate production before exercise (B/A), and the lactate production difference between after exercise and after rest was divided by lactate production after rest; this was defined as the clearance rate ((B − C)/B). Values in the same row with different superscript letters (^a^, ^b^, ^c^) differed significantly at *p* < 0.05 by one-way analysis of variance (ANOVA).

**Table 3:** Effects of exercise and probiotic intervention on clinical biochemical analysis at the end of the experiment.

Parameter	Sedentary	Exercise	OLP-01	Exercise + OLP-01
AST (U/L)	64 ± 9 ^a^	92 ± 24 ^b^	69 ± 10 ^a^	62 ± 10 ^a^
ALT (U/L)	38 ± 5	43± 9	37 ± 4	37 ± 2
CK (U/L)	136 ± 41 ^a^	210 ± 69 ^b^	143 ± 47 ^a^	141 ± 37 ^a^
GLU (mg/dL)	147 ± 24	144 ± 23	148 ± 22	151 ± 29
CREA (mg/dL)	0.41± 0.03	0.40 ± 0.04	0.40 ± 0.03	0.41 ± 0.02
BUN (mg/dL)	23.1 ± 1.6	22.9 ± 1.5	23.0 ± 1.4	23.1 ± 1.4
UA (mg/dL)	2.41 ± 0.4	2.35 ± 0.6	2.41 ± 0.5	2.48 ± 0.4
TC (mg/dL)	136 ± 22	133 ± 27	130 ± 20	128 ± 13
TG (mg/dL)	147 ± 30	164 ± 26	140 ± 19	143 ± 19
ALB (g/dL)	3.2 ± 0.1	3.1 ± 0.2	3.1 ± 0.2	3.1 ± 0.1
TP (g/dL)	5.5 ± 0.3	5.4 ± 0.4	5.3 ± 0.2	5.3 ± 0.3

Data are expressed as the mean ± SD in each group. Values in the same row with different superscript letters (^a^, ^b^) differed significantly at *p* < 0.05 by one-way ANOVA. AST, aspartate aminotransferase; ALT, alanine transaminase; NH_3_, ammonia; CK, creatine kinase; GLU, glucose; CREA, creatinine; BUN, blood urea nitrogen; UA, uric acid; TC, total cholesterol; TG, triacylglycerol; ALB, albumin; TP, total protein.

**Table 4:** Effects of exercise and probiotic interventions on complete blood count analysis.

Parameter	Sedentary	Exercise	OLP-01	Exercise + OLP-01
WBC (10^3^/μL)	12.2 ± 4.5	12.4 ± 2.4	12.8 ± 5.3	11.5 ± 3.1
Neu (%)	17.7 ± 8.1	17.3± 7.5	18.1 ± 10.1	17.2 ± 5.2
Lym (%)	81.2 ± 9.2	80.4 ± 9.1	81.4 ± 11	81.2 ± 5.5
Mono (%)	0.74 ± 1.3	1.34 ± 1.7	0.2 ± 0.1	0.54 ± 0.4
Eosi (%)	0.14 ± 0.2 ^a^	0.64± 0.8 ^a,b^	0.21 ± 0.2 ^a,b^	0.78 ± 0.8 ^b^
Baso (%)	0.19 ± 0.1	0.28 ± 0.2	0.16 ± 0.1	0.27 ± 0.3
Platelet (10^3^/μL)	1216 ± 283 ^b^	1646 ± 332 ^c^	957 ± 124 ^a^	1009 ± 145 ^a,b^
PLR	123 ± 22 ^a^	187 ± 37 ^b^	108 ± 18 ^a^	117 ± 17 ^a^
NLR	0.21 ± 0.08	0.26 ± 0.12	0.18 ± 0.06	0.20 ± 0.07

Data are expressed as the mean ± SD in each group. Values in the same row with different superscript letters (^a^, ^b^) differed significantly at *p* < 0.05 by one-way ANOVA. WBC, white blood cells; Neu, neutrophils; Lym, lymphocytes; Mono, monocytes; Eosi, eosinophils; Baso, basophils; PLR, platelet/lymphocyte ratio; NLR, neutrophil/lymphocyte ratio.

**Table 5:** The effects of exercise and probiotics on fecal short-chain fatty acid content.

SCFAs	Sedentary	Exercise	OLP-01	Exercise + OLP-01
acetic acid	3.12 ± 0.34 ^a^	3.75± 0.54 ^a,b^	3.18 ± 0.41 ^a^	4.22 ± 0.39 ^b^
propionic acid	0.49 ± 0.08 ^a^	0.54± 0.07 ^a^	0.52 ± 0.08 ^a^	0.71 ± 0.11 ^b^
isobutyric acid	0.02 ± 0.003 ^a^	0.02 ± 0.004 ^a^	0.02 ± 0.006 ^a^	0.03 ± 0.006 ^a^
butyric acid	0.53 ± 0.15 ^a^	0.85 ± 0.28 ^b, c^	0.59 ± 0.24 ^a,b^	1.16 ± 0.18 ^c^
valeric acid	0.053 ± 0.02	0.029 ± 0.01	0.025 ± 0.02	0.048 ± 0.02

Data are expressed as the mean ± SD in each group. Values in the same row with different superscript letters (^a^, ^b^, ^c^) differed significantly at *p* < 0.05 by one-way ANOVA. The unit of indicated SCFAs is mM.
